# Supplementary material for: Causal Relationship between Aging and Anorexia Nervosa: A White-Matter-Microstructure-Mediated Mendelian Randomization Analysis
Source: Biomedicines. 2024 Aug 16;12(8):1874. doi: 10.3390/biomedicines12081874 (PMC11351342; doi:10.3390/biomedicines12081874)
Supplement: Supplementary file 1 [file biomedicines-12-01874-s001.zip › Supplementary Figure/Figure S4/Singe SNP plot of MR analysis for TL to ubm-b-2019.pdf]

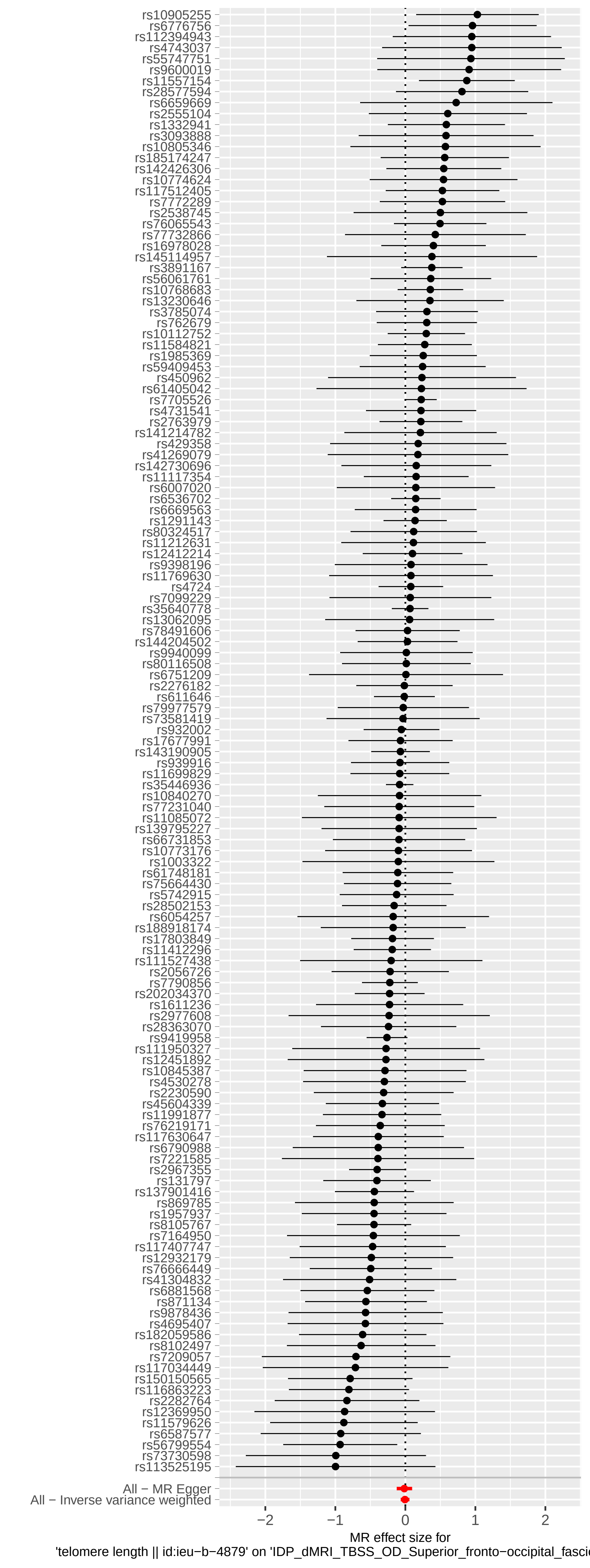

rs10905255
rs6776756
rs112394943
rs4743037
rs55747751
rs9600019
rs11557154
rs28577594
rs6659669
rs2555104
rs1332941
rs3093888
rs10805346
rs185174247
rs142426306
rs10774624
rs117512405
rs7772289
rs2538745
rs76065543
rs77732866
rs16978028
rs145114957
rs3891167
rs56061761
rs10768683
rs13230646
rs3785074
rs762679
rs10112752
rs11584821
rs1985369
rs59409453
rs450962
rs61405042
rs7705526
rs4731541
rs2763979
rs141214782
rs429358
rs41269079
rs142730696
rs11117354
rs6007020
rs6536702
rs6669563
rs1291143
rs80324517
rs11212631
rs12412214
rs9398196
rs11769630
rs4724
rs7099229
rs35640778
rs13062095
rs78491606
rs144204502
rs9940099
rs80116508
rs6751209
rs2276182
rs611646
rs79977579
rs73581419
rs932002
rs17677991
rs143190905
rs939916
rs11699829
rs35446936
rs10840270
rs77231040
rs11085072
rs139795227
rs66731853
rs10773176
rs1003322
rs61748181
rs75664430
rs5742915
rs28502153
rs6054257
rs188918174
rs17803849
rs11412296
rs111527438
rs2056726
rs7790856
rs202034370
rs1611236
rs2977608
rs28363070
rs9419958
rs111950327
rs12451892
rs10845387
rs4530278
rs2230590
rs45604339
rs11991877
rs76219171
rs117630647
rs6790988
rs7221585
rs2967355
rs131797
rs137901416
rs869785
rs1957937
rs8105767
rs7164950
rs117407747
rs12932179
rs76666449
rs41304832
rs6881568
rs871134
rs9878436
rs4695407
rs182059586
rs8102497
rs7209057
rs117034449
rs150150565
rs116863223
rs2282764
rs12369950
rs11579626
rs6587577
rs56799554
rs73730598
rs113525195
All − MR Egger
All − Inverse variance weighted
−2
−1
0
1
2
MR effect size for
'telomere length || id:ieu−b−4879' on 'IDP_dMRI_TBSS_OD_Superior_fronto−occipital_fasci
